# Antiviral Activity of Selected Lamiaceae Essential Oils and Their Monoterpenes Against SARS-Cov-2

**DOI:** 10.3389/fphar.2022.893634

**Published:** 2022-05-02

**Authors:** Sanja Ćavar Zeljković, Ermin Schadich, Petr Džubák, Marián Hajdúch, Petr Tarkowski

**Affiliations:** ^1^ Centre of the Region Haná for Biotechnological and Agricultural Research, Department of Genetic Resources for Vegetables, Medicinal and Special Plants, Crop Research Institute, Olomouc, Czechia; ^2^ Centre of Region Haná for Biotechnological and Agricultural Research, Czech Advanced Technology and Research Institute, Palacky University, Olomouc, Czechia; ^3^ Institute of Molecular and Translational Medicine, Faculty of Medicine and Dentistry, Palacky University, Olomouc, Czechia; ^4^ Institute of Molecular and Translational Medicine, Czech Advanced Technology and Research Institute, Palacky University, Olomouc, Czechia

**Keywords:** essential oil, monoterpene, carvacrol, carvone, pulegone, antiviral activity, SARS-CoV-2, 1,8-cineol

## Abstract

This study presents the very first report on the *in vitro* antiviral activity of selected essential oils of Lamiaceae plant species and their monoterpenes against severe acute respiratory syndrome coronavirus 2 (SARS-CoV-2). Nineteen essential oils were obtained by hydrodistillation of dried plant material, and their monoterpene profiles were determined. In addition, the exact concentrations of each monoterpene that were found at a significant level were defined. Both essential oils and their monoterpene components were tested for cytotoxic and antiviral activity against SARS-CoV-2 in infected Vero 76 cells. The results showed that the essential oils of four *Mentha* species, i.e., *M. aquatica* L. cv. Veronica, *M. pulegium* L., *M. microphylla* K.Koch, and *M. x villosa* Huds.*,* but also *Micromeria thymifolia* (Scop.) Fritsch and *Ziziphora clinopodioides* Lam., and five different monoterpenes, i.e., carvacrol, carvone, 1,8-cineol, menthofuran, and pulegone, inhibited the SARS-CoV-2 replication in the infected cells. However, the antiviral activity varied both among essential oils and monoterpenes. Carvone and carvacrol exhibited moderate antiviral activity with IC_50_ concentrations of 80.23 ± 6.07 μM and 86.55 ± 12.73 μM, respectively, while the other monoterpenes were less active (IC_50_ > 100.00 μM). Structure-activity relations of related monoterpenes showed that the presence of keto and hydroxyl groups is associated with the activity of carvone and carvacrol, respectively. Furthermore, the carvone-rich essential oil of *M. x villosa* had the greatest activity among all active essential oils (IC_50_ 127.00 ± 4.63 ppm) while the other active oils exhibited mild (140 ppm < IC_50_ < 200 ppm) to weak antiviral activity (IC_50_ > 200 ppm). Both essential oils and monoterpenes showed limited or no cytotoxicity against Vero 76 cells. Hierarchical cluster analysis showed that the differences in the antiviral activity of essential oils were directly attributed to the antiviral efficacies of their particular single monoterpenes. The findings presented here on the novel antiviral property of plant essential oils and monoterpenes might be used in the development of different measures against SARS-CoV-2.

## Introduction

In 2020, the world health organization (WHO) declared the outbreak of the highly contagious coronavirus disease 2019 (COVID-19) is a pandemic (WHO, 2020). Such disease spread was due to the introduction of its etiological agent, the highly virulent severe acute respiratory syndrome coronavirus 2 (SARS-CoV-2) into immunologically naive populations (Leung et al., 2020). Since then, to mitigate the severity and spread of COVID-19, both scientific communities and health organizations are focused on the development of effective prophylactic and therapeutic measures (Canedo-Marroquín et al., 2020; Kampf et al., 2020). Both novel synthetic compounds and natural products have been included in strategies for the development of novel antiviral drug candidates against the SARS-CoV-2 virus (Mei and Tan, 2021; Singla et al., 2021). Among the different natural products considered for the development of effective antiviral drugs are also different classes of plant terpenes (Diniz et al., 2021; Singla et al., 2021). Terpenes are usually isolated from plants as the natural mixture called essential oils that have been used for different remedies against different non-infectious and infectious diseases since ancient times (Dzubak et al., 2006; Jamshidi-Kia et al., 2018). Due to this, the pharmacological properties of essential oils and their terpene components have been analyzed extensively studied (Angane et al., 2022), and their different antimicrobial, antiviral, antioxidant, anti-inflammatory, and anticancer activities were reported (Amorati et al., 2013; Wani et al., 2021; Esmaeili et al., 2022; Singh et al., 2022).

The antiviral activity of plant essential oils is attributed to the content and composition of monoterpenes and sesquiterpenes (Das et al., 2021; Kim, 2021; Salem and Ezzat, 2021; Wani et al., 2021). Some essential oils are very active against human herpesvirus (HSV), influenza A virus (H1N1), avian influenza A virus (H5N1), and human immunodeficiency virus (HIV) (Das et al., 2021; Wani et al., 2021). Furthermore, both essential oils and their monoterpene components might also have antiviral activity against SARS-CoV-2 as the molecular docking studies showed that different plant monoterpenes and sesquiterpenes might bind and interfere with the functions of different proteins of SARS-Cov-2 virus including main protease, endoribonuclease, ADP ribose phosphatase, RNA-dependent RNA polymerase and spike protein (Silva et al., 2020; Das et al., 2021; Kim, 2021), and human cell proteins important for viral internalization and replication including, angiotensin-converting enzyme and cellular proteases, transmembrane serine protease 2, cathepsin B, and cathepsin L (Silva et al., 2020; Panikar et al., 2021; Salem and Ezzat, 2021; Torres Neto et al., 2021).

Therefore, in a continuation of our research on essential oils and monoterpenes from medicinal and aromatic plants of the Lamiaceae family, we were encouraged to test, for the very first time, the activity of selected plant essential oils and their monoterpenes for activity against SARS-Cov-2. The selection of the essential oils of different plant species and chemotypes was based on their use in different aspects of traditional medicine, but also in the food and pharmaceutical industry (Ćavar et al., 2008; Ćavar Zeljković and Maksimović, 2015; Ćavar Zeljković et al., 2021b). The chemical characterization of oils was performed by profiling their monoterpene contents and by measuring the exact concentrations of each monoterpene component. Essential oils and their major monoterpene components were tested for cytotoxic and antiviral activities, and obtained results were correlated with concentrations of active components.

## Materials and Methods

### Plant Material and Essential Oil Isolation

All plants used in this study were grown in the experimental field of the Crop Research Institute in Olomouc, Department of Genetic Resources for Vegetables, Medicinal and Special Plants, Olomouc, Czechia including *Mentha x villosa* Huds. (MVI), *Mentha pulegium* L. (MPU), *Mentha microphylla* K.Koch (MMI), *Mentha aquatica* L. cv. Veronica (MAV), *Mentha x piperita* L. (MPI), *Mentha x piperita* L. cv. Bergamot (MPB), *Mentha x piperita* L. cv. Citrata (MPC), *Mentha x piperita* L. cv. Perpeta (PPP), *Thymus vulgaris* L. (chemotypes TVU 1 and TVU2), *Thymus x citriodorus* (Pers.) Schreb. (TCI), *Lavandula angustifolia* Mill. (chemotypes LAN1, LAN2, and LAN3), *Saturaja montana* L. (chemotypes SMO1 and SMO2), *Micromeria thymifolia* (Scop.) Fritsch (MTY), *Hyssopus officinalis* L. (HOF), and *Ziziphora clinopodioides* Lam. (ZCL). The plants were harvested during the early flowering stages and dried in the dark under airflow, and leaves were crushed and homogenized. Essential oils were obtained by hydrodistillation of 20 g of dried plant material (1.5 h) on a Clevenger apparatus. The oils were kept at 4°C until analysis.

### GC/MS Analysis

Essential oils (20 μl) were dissolved in 1 ml of *n*-hexane containing *n*-undecane as internal standard (Merck Company, Czechia), and the composition of essential oil constituents was analyzed *via* GC–MS using an Agilent 7890A gas chromatograph fitted with a fused silica HP-5MS UI (5% phenyl methylsiloxane) capillary column (30 m × 0.25 mm, 0.25 μm film thickness) coupled to an Agilent 5975C mass selective detector. All details about GC/MS conditions were described earlier (Ćavar Zeljković et al., 2021a; Ćavar Zeljković et al., 2021b). The identification of the constituents was accomplished by visual interpretation, a comparison of their retention indices and mass spectra with literature data (Adams, 2007), and a computer library search (Mass Finder 4 Computer Software).

**FIGURE 1:**
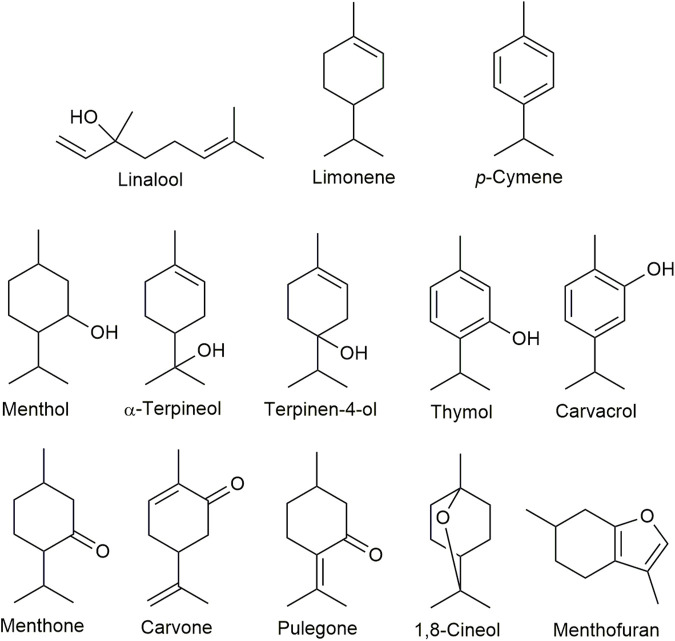
Structures of monoterpenes investigated in this study.

The standard solutions of selected monoterepenes (*p*-cymene, 1,8-cineole, limonene, linalool, menthone, menthofuran, menthol, terpinen-4-ol, α-terpineol, pulegone, carvone, thymol, and carvacrol) were dissolved in DMSO (dimethyl sulfoxide, Merck Company, Czechia) in the concentration of 100 mg/ml. Further dilutions were prepared in *n*-hexane (containing *n*-undecane as the internal standard) in a concentration range 25–1,000 μg/ml. All standards were purchased from Merck Company (Czechia). The concentrations of these compounds in oils were calculated *via* external standard calibration. Mass spectra were measured in SIM (single ion monitoring) mode, monitoring specific qualifier (underlined) and quantifier ions for each particular compound, i.e., *p*-cymene (91, 134, 119, 134), 1,8-cineole (43, 81, 108, 154), limonene (68, 93, 121, 136), linalool (43, 71, 93, 154), menthone (41, 69, 112, 154), menthofuran (79, 108, 150), menthol (71, 95, 123, 138), terpinene-4-ol (71, 93, 111, 154), α-terpineol (59, 93, 121, 136), pulegone (67, 81, 109, 152), carvone (54, 82, 93, 150), thymol (91, 115, 135, 150), and carvacrol (91, 115, 135, 150). The structures of these compounds are presented in Figure 1. Measurements are performed in three replicates.

### Cell and Viral Cultures

VERO 76 cells were obtained from the American Type Culture Collection (ATCC). Cells were in full Dulbecco growth medium [10% inactivated fetal calf serum (FCS), streptomycin (100 μg/ml), and penicillin (100  IU/ml)] at 37 °C at 5% CO_2_. The SARS-CoV-2 isolate from the first Czech patient was obtained from The National Institute of Public Health, Czechia (Klempt et al., 2021).

### Cytotoxicity Assay

The cytotoxicity of monoterpenes and essential oils on Vero 76 cells and relevant IC_50_ values were determined by using an established MTS tetrazolium assay, as described in our previous study (Schadich et al., 2020). Monoterpenes were tested in four-fold serial dilutions within 2,000-1.95 μM concentration range while essential oils were tested four-fold serial dilutions within 2,000-in-0.49 ppm concentration range. Both monoterpene standards and essential oils were dissolved in dimethyl sulfoxide. Cytotoxicity was considered for the IC_50_ values below 1 mM.

### Antiviral Assay

For the antiviral assay, 100 μl of suspension of VERO 76 cells (1 × 10^5^ cells/ml) in full Dulbecco growth medium were seeded into the wells of 96 well plate for overnight incubation at 37 °C in a 5% CO_2_ humidified incubator. The cells were infected by inoculation media [Dulbecco growth medium supplemented with 1% FCS, streptomycin (100 μg/ml), and penicillin (100  IU/ml)], with the virus at 0.02 MOI in 2 h incubation as described in Shin et al. (2020). The wells were washed twice by inoculation media and monoterpenes or essential oils in concentration ranges were added to the wells. Monoterpenes were tested in four-fold serial dilutions within 1,000–4.91 μM concentration range while essential oils were tested in four-fold serial dilutions within 250-in-0.98 ppm concentration range. The exceptions were concentrations ranges for cytotoxic monoterpenes and plant extracts, and they were adjusted to safe concentrations ranges. The viral control reactions received 0.5% DMSO while the positive reactions received 40 μM chloroquine as a reference *in vitro* antiviral compound. Mock-infected controls received only 0.5% DMSO. After 72 h incubation, the cell viability was determined by methylthiazolyldiphenyl-tetrazolium bromide (MTT) assay received 10 μl of MTT solution (5 mg/ml in phosphate-buffered saline) was added into each reaction well. Following a 3 h incubation time at 37 °C, the media was discarded and formazan crystals were dissolved in 100 μl of a 1:2 mixture of DMSO and ethanol. The absorption of wells was measured at 590 nm. The antiviral effective concentration (EC_50_) was expressed as the concentration of monoterpenes and plant extracts that provided 50% inhibition of the SARS-CoV-2 induced destruction of infected cells. The percent of SARS-CoV-2 inhibition was calculated by the following formula:

$$\frac{{\left(ODT\right)}_{SARS-Cov-2}-{\left(ODc\right)}_{SARS-Cov-2}}{{\left(ODc\right)}_{MocK}-{\left(ODc\right)}_{SARS-Cov-2}}x\;100\%$$

where (ODT)_SARS-Cov-2_ is the absorbance of the test sample, (ODc)_SARS-CoV-2_ is the absorbance of the virus-infected control (no monoterpene or essential oil), and (ODc)_MocK_ is the absorbance of the mock-infected control. The EC_50_ was determined by non-linear regression analysis of SARS-CoV-2 inhibition using GraphPad Prism 5 software. Only the values with an R factor >0.8 were selected. The experiments were done three times with three technical replicates for each tested concentration of monoterpenes and essential oils.

### Statistical Analysis

Experimental results were presented in tables and graphs as the mean ± standard deviation of three independent replications. Data obtained were subjected to variance analysis (ANOVA) and the Newman–Keuls post-hoc test was carried out to identify significant differences between samples, using TIBCO-STATISTICA 14.0.0 software package. Mean values with *p* < 0.01 were considered statistically significant. Pearson correlations were performed to observe the correlation between the essential oil profile and cytotoxic and antiviral activity at the level of significance *p* < 0.05. Heatmaps and correlation analysis were performed in Orange data mining software v.3.31.1. Multiple hypothesis adjustment of all *p* values was carried out using the Bonferroni correction.

## Results

### Essential Oil Composition

**FIGURE 2:**
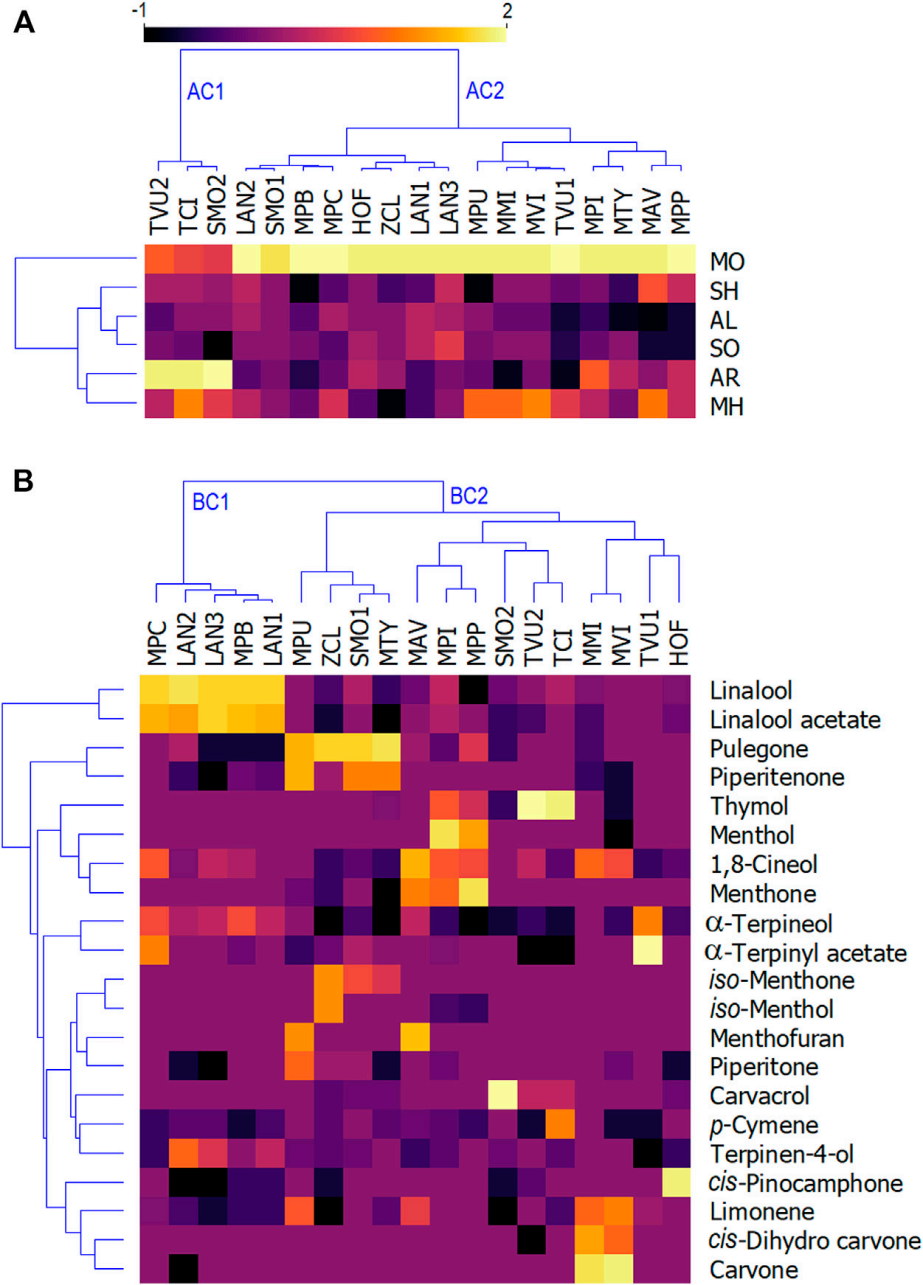
Heatmaps of the levels of volatile compounds in investigated essential oils. **(A)** classes of essential oil volatiles; **(B)** the most abundant terpenes in the oils. MAV—*Mentha aquatica* “Veronica”; MPU—*Mentha pulegium*; MMI—*Mentha microphylla*; MVI—*Mentha vilosa*; MPI—*Mentha x piperita*; MPB—*Mentha x piperita* “Bergamot”; MPP—*Mentha x piperita* “Perpeta”; MPC—*Mentha x piperita* “Citrata”; TVU1—*Thymus vulgaris* 1; TVU2—*Thymus vulgaris 2*; TCI—*Thumys x citriodorus*; LAN1—*Lavandula augustifolia* 1; LAN2—*Lavandula augustifolia* 2; LAN3—*Lavandula augustifolia* 3; SMO1—*Satureja montana* 1; SMO2—*Satureja montana* 2; MTY—*Micromeria thymifolia*; HOF—*Hyssopus officinalis*; ZCL—*Ziziphora clinopodioides;* MH—monoterpene hydrocarbons; MO—oxygenated monoterpenes; SH—sesquiterpene hydrocarbons; SO—oxygenated sesquiterpenes; AL—aliphatic compounds; AR—aromatic compounds.

Profiling of essential oils of selected Lamiaceae plants was performed *via* GC/MS analysis where the majority of volatile constituents were identified and quantified (Supplementary Table S1). Altogether, 90 compounds were identified in 19 samples, representing 70.4%–99.1% of the total compounds. Figure 2 represents the heatmaps of the levels of different classes of chemical constituents in investigated essential oils (Figure 2A), as well as the main constituents of the oils (Figure 2B), which are both clustered into two main groups (clusters). For better visualization, the percentage content of both classes and the major compounds are transformed into logarithmic values (log_10_). When classes of volatiles were considered (Figure 2A), the essential oils were clustered into two main groups which are mainly separated by the content of aromatic compounds and oxygenated monoterpenes. Group AC1 contained oils with significant levels of aromatic monoterpenes (*T. vulgaris* 2, *T. x citriodorus*, and *S. montana* 2), while group AC2 contained 14 essential oils which have significant levels of oxygenated monoterpenes. Furthermore, essential oils were clustered according to the logarithmic value of the percentage content of their most abundant compounds (Figure 1B). Again, the oils were separated into two main clusters. Cluster BC1 consisted of essential oils rich in linalool and linalool acetate, while cluster BC2 contained all other essential oils. Cluster BC2 was more complex and contained two subclusters. One subcluster contained the essential oils that have significant levels of pulegone and piperitenone, while the other subcluster contained the essential oils rich in aromatic thymol and carvacrol (*Thymus* and *Satureja* species), and other components (Figure 2B).

Since the bioactivity of natural extracts is dependent on the concentration of active components (Prusinowska and Smigielski, 2015; Ćavar Zeljković et al., 2021a), the levels of the main constituents of each oil were quantified via the external calibration method. The essential oils of *T. vulgaris* 2 (TVU2) and *T. x citriodus* (TCI) are extremely rich in thymol, reaching 807.28 ± 4.41 and 866.75 ± 8.99 mg/ml, respectively (Table 1, Supplementary Table S1). In addition, one chemotype of *S. montana* (SMO2) mainly contains carvacrol (901.32 ± 3.55 mg/ml). Other investigated essential oils show higher heterogeneity, where the concentration of the main component was not exceeding 260 mg/ml (Table 1).

**TABLE 1:** Concentrations of the main constituents of investigated essential oils.

Essential oil	Compound (mg/ml)												
	*p*-Cymene	Thymol	Carvacrol	Limonene	1,8-Cineol	Linalool	Menthone	Menthofuran	Menthol	Terpinen-4-ol	α-Terpineol	Pulegone	Carvone
MAV	2.45 ± 0.08^c^	nd	nd	15.03 ± 0.42^c^	96.75 ± 1.73^a^	4.17 ± 0.25^g^	68.48 ± 1.18^b^	81.90 ± 2.01^a^	nd	2.62 ± 0.03^i^	17.68 ± 0.49^f^	7.04 ± 0.48^fg^	nd
MPU	nd	nd	nd	29.84 ± 0.26^b^	nd	nd	4.74 ± 0.21^d^	43.52 ± 1.02^b^	nd	4.53 ± 0.06^f^	nd	134.74 ± 3.45^c^	nd
MMI	nd	nd	nd	29.30 ± 1.24^b^	24.11 ± 1.32^b^	4.28 ± 0.03^g^	nd	nd	nd	nd	1.45 ± 0.35^i^	5.00 ± 3.46^fg^	227.23 ± 2.84^b^
MVI	1.18 ± 0.07^d^	nd	nd	52.24 ± 0.90^a^	16.52 ± 0.47^d^	1.72 ± 0.07^g^	nd	nd	1.43 ± 0.05^c^	nd	3.17 ± 0.06^i^	nd	256.76 ± 3.13^a^
MPI	1.79 ± 0.09^cd^	63.95 ± 2.34^c^	nd	3.31 ± 0.47^f^	19.69 ± 1.63^c^	8.23 ± 0.06^f^	32.11 ± 1.71^c^	nd	195.53 ± 2.54^a^	3.17 ± 0.11^h^	3.42 ± 0.07^i^	3.25 ± 0.08^g^	nd
MPB	1.25 ± 0.08^d^	nd	nd	1.54 ± 0.22^g^	5.00 ± 0.09^f^	127.25 ± 3.50^d^	nd	nd	nd	nd	34.47 ± 1.17^c^	1.55 ± 0.22^g^	nd
MPP	1.65 ± 0.09^d^	21.90 ± 1.51^d^	nd	4.61 ± 0.20^e^	15.93 ± 0.25^d^	1.68 ± 0.13^g^	181.3 ± 1.56^a^	nd	76.34 ± 1.86^b^	nd	2.32 ± 0.16^i^	13.32 ± 1.02^e^	nd
MPC	1.86 ± 0.08^c^	nd	nd	3.87 ± 0.36^gf^	24.14 ± 2.57^b^	172.55 ± 1.53^b^	nd	nd	nd	2.64 ± 0.05^i^	53.13 ± 1.68^b^	nd	nd
TVU1	1.44 ± 0.07^d^	nd	nd	8.66 ± 0.17^d^	2.07 ± 0.04^fg^	6.93 ± 0.15^f^	nd	nd	nd	1.85 ± 0.08^j^	158.66 ± 3.16^a^	nd	nd
TVU2	1.27 ± 0.07^d^	866.75 ± 8.99^a^	27.76 ± 0.65^c^	nd	7.92 ± 0.16^eg^	17.92 ± 0.39^e^	nd	nd	nd	6.14 ± 0.06^e^	10.07 ± 0.25^h^	1.23 ± 0.20^g^	nd
TCI	41.26 ± 0.91^b^	807.28 ± 4.41^b^	30.67 ± 0.68^b^	2.86 ± 0.05^fg^	3.23 ± 0.13^fg^	10.73 ± 0.51^f^	nd	nd	nd	4.44 ± 0.07^f^	3.52 ± 0.03^i^	nd	nd
LAN1	1.38 ± 0.23^d^	nd	nd	1.87 ± 0.06^g^	3.24 ± 0.13^fg^	127.37 ± 3.58^d^	nd	nd	nd	8.67 ± 0.11^c^	14.79 ± 0.17^g^	2.22 ± 0.04^fg^	nd
LAN2	2.24 ± 0.14^cd^	nd	nd	2.67 ± 0.06^fg^	4.15 ± 0.15^fg^	232.48 ± 2.81^a^	nd	nd	nd	41.13 ± 0.49^a^	18.00 ± 0.96^ef^	8.39 ± 0.44^f^	1.88 ± 0.17^c^
LAN3	2.14 ± 0.05^cd^	nd	nd	1.74 ± 0.05^g^	7.76 ± 0.15^e^	164.05 ± 1.59^c^	nd	nd	nd	16.83 ± 0.31^b^	21.48 ± 0.73^d^	1.68 ± 0.19^g^	nd
SMO1	nd	nd	nd	nd	nd	3.63 ± 0.14^g^	nd	nd	nd	2.05 ± 0.09^j^	2.07 ± 0.16^i^	52.60 ± 0.92^d^	nd
SMO2	4.83 ± 0.25^b^	nd	901.32 ± 3.55^a^	1.64 ± 0.05^g^	3.28 ± 0.07^fg^	7.60 ± 0.17^f^	nd	nd	nd	7.36 ± 0.06^d^	4.62 ± 0.14^i^	nd	nd
MTY	1.84c ± 0.22^d^	9.05 ± 0.18^e^	nd	2.81f ± 0.10^g^	1.68 ± 0.14^g^	2.63 ± 0.06^f^	1.68 ± 0.11^e^	nd	nd	5.84 ± 0.07^e^	2.15 ± 0.05^i^	221.94 ± 1.49^a^	nd
HOF	2.35 ± 0.09^cd^	nd	nd	1.15 ± 0.05^g^	1.73 ± 0.06^g^	3.79 ± 0.10^f^	nd	nd	nd	nd	3.33 ± 0.06^i^	nd	nd
ZCL	2.06 ± 0.05^cd^	nd	nd	1.52 ± 0.07^g^	2.12 ± 0.18^g^	3.36 ± 0.21^f^	nd	nd	nd	3.71 ± 0.19^g^	0.21 ± 0.05^j^	196.54 ± 5.61^b^	2.37 ± 0.06^c^

Essential oils of MAV, Mentha aquatica “Veronica”; MPU, Mentha pulegium; MMI, Mentha microphylla; MVI, Mentha vilosa; MPI, Mentha x piperita; MPB, Mentha x piperita “Bergamot”; MPP, Mentha x piperita “Perpeta”; MPC, Mentha x piperita “Citrata”; TVU1, Thymus vulgaris 1; TVU2, Thymus vulgaris 2; TCI, Thumys x citriodorus; LAN1, Lavandula augustifolia 1; LAN2, Lavandula augustifolia 2; LAN3, Lavandula augustifolia 3; SMO1, Satureja montana 1; SMO2, Satureja montana 2; MTY, Micromeria thymifolia; HOF, Hyssopus officinalis; ZCL, Ziziphora clinopodioides; nd, not detected.

Data represent means of three replicates (± standard deviation). Values within one column followed by the same letter do not differ significantly after factorial analysis of variance (ANOVA) *post hoc* Newman–Keuls analysis at a significance level of *p* < 0.01.

### Cytotoxicity of Selected Monoterpenes and Essential Oils

Among thirteen tested compounds, only the three monoterpenes, i.e., carvacrol, menthofuran, and thymol, exhibited low cytotoxic activity against Vero 76 cells. The other monoterpenes were not cytotoxic to Vero 76 cells (Table 2). The IC_50_ values of carvacrol, menthofuran, and thymol against Vero 76 cells were 455.00 ± 31.11, 505.57 ± 76.43, and 508.60 ± 31.48 μM, respectively. Nine essential oils also exhibited low cytotoxic activity against Vero 76 cells while the other ten were not cytotoxic (Table 3). However, among these nine cytotoxic essential oils, the cytotoxicity to Vero 76 cells varied as the four oils, i.*e., M. x piperita*, *S. mont*ana 2 (carvacrol chemotype), *T. x citriodorus*, and *T. vulgaris* 2 (thymol chemotype) had the cytotoxicity activity greater than that of the other five cytotoxic natural products with IC_50_ smaller than concentration value of 500 ppm (Table 3).

**TABLE 2:** Cytotoxic and antiviral activity of selected monoterpenes.

Compound	IC_50_ (μM)	EC_50_ (μM)	TI
	Vero 76 cells	SARS-Cov2	
Carvacrol	455.00 ± 31.11^a^	80.23 ± 6.07^d^	6.23
Carvone	NA	86.55 ± 12.73^d^	>5.78
1,8-Cineol	NA	128.93 ± 14.98^c^	>3.88
*p*-Cymene	NA	NA	NA
Limonene	NA	NA	NA
Linalool	NA	NA	NA
Menthofuran	505.57 ± 76.43^a^	154.37 ± 16.33^b^	3.28
Menthol	NA	NA	NA
Menthone	NA	NA	NA
Pulegone	NA	195.70 ± 17.12^a^	>2.55
α-Terpineol	NA	NA	NA
Terpinen-4-ol	NA	NA	NA
Thymol	508.60 ± 31.48^a^	LA	NA
Chloroquine	NA	7.57 ± 1.18^e^	>13.21

IC_50_ and EC_50_ values are means (±SD) of three independent biological replicates with three technical replicates. The therapeutic index (TI) is the ratio of the average IC_50_ value of the relevant compound and natural plant extract against Vero cells to its IC_50_ value against SARS-Cov2. Cytotoxicity was considered for the IC_50_ values below 1 mM. NA and LA denote no activity and limited activity, respectively. Values within one column followed by the same letter do not differ significantly after factorial analysis of variance (ANOVA) *post hoc* Newman–Keuls analysis at a significance level of *p* < 0.01.

**TABLE 3:** Cytotoxic and antiviral activity of essential oils.

Code	Essential oil	IC_50_ (ppm)	EC_50_ (ppm)	TI
	Plant	Vero 76 cells	SARS-Cov2	
MAV	*Mentha aquatica* “Veronica”	516.53 ± 34.02^b^	189.73 ± 32.03^b^	2.72
MPU	*Mentha pulegium*	NA	148.50 ± 27.59^b^	>13.47
MPI	*Mentha x piperita*	423.13 ± 20.49^b^	NA	NA
MPB	*Mentha x piperita* “Bergamot”	NA	NA	NA
MPP	*Mentha x piperita* “Perpeta”	NA	LA	NA
MMI	*Mentha microphylla*	1,400.00 ± 263.55^a^	179.33 ± 23.97^b^	7.81
MVI	*Mentha vilosa*	1,177.27 ± 219.62^a^	127.00 ± 4.63^b^	9.27
MPC	*Mentha x piperita* “Citrata”	NA	LA	NA
TVU1	*Thymus vulgaris* 1	1,271.00 ± 193.53^a^	NA	NA
TVU2	*Thymus vulgaris* 2	258.80 ± 26.66^b^	NA	NA
TCI	*Thumys x citriodorus*	265.27 ± 56.68^b^	NA	NA
LAN1	*Lavandula augustifolia* 1	NA	NA	NA
LAN2	*Lavandula augustifolia* 2	NA	NA	NA
LAN3	*Lavandula augustifolia* 3	NA	NA	NA
SMO1	*Satureja montana* 1	NA	NA	NA
SMO2	*Satureja montana* 2	189.57 ± 21.02^b^	LA	NA
MTY	*Micromeria thymifolia*	NA	297.10 ± 48.31^a^	6.73
HOF	*Hyssopus officinalis*	NA	NA	NA
ZCL	*Ziziphora clinopodioides*	1,110.43 ± 128.26^a^	339.37 ± 63.56^a^	3.27

The reported IC_50_ and EC_50_ values are means (±SD) of three independent biological replicates with three technical replicates. The therapeutic index (TI) is the ratio of the average IC_50_ value of the relevant compound and natural plant extract against Vero cells to its IC_50_ value against SARS-Cov2. NA and LA denote no activity and limited activity, respectively. Values within one column followed by the same letter do not differ significantly after factorial analysis of variance (ANOVA) *post hoc* Newman–Keuls analysis at a significance level of *p* < 0.01.

### Antiviral Activity of Selected Monoterpenes and Essential Oils

**FIGURE 3:**
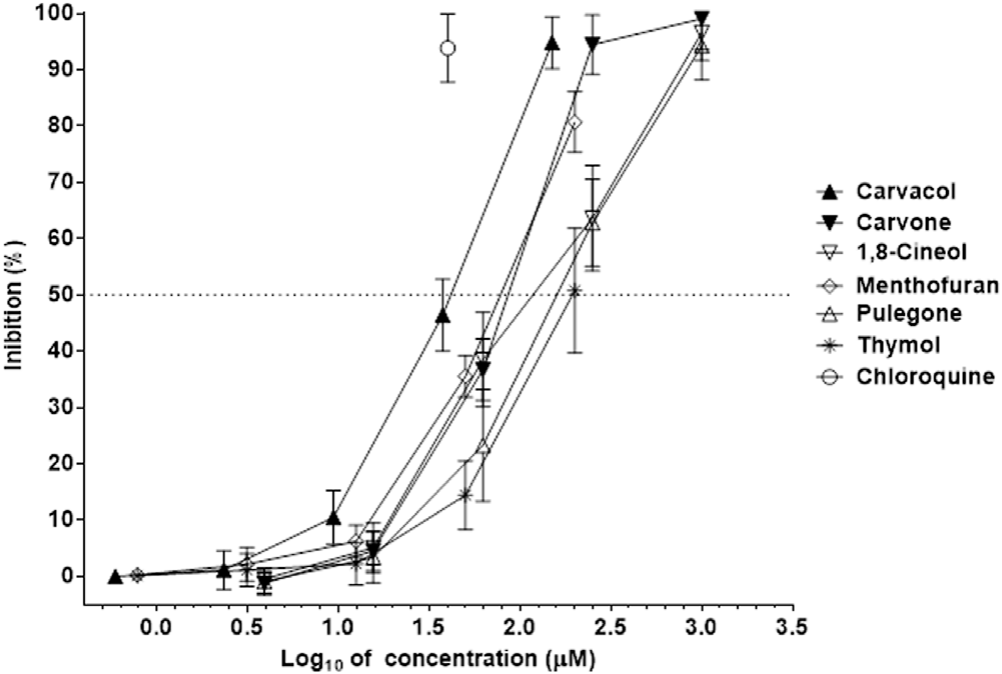
Inhibition of SARS-CoV-2 by monoterpenes. The inhibition rates of SARS-CoV-2 of monoterpenes in infected Vero 76 cells were determined in 72 h incubation assays. Chloroquine was used as a reference antiviral compound. Data points represent the means (±SD) of three independent biological replicates with three technical replicates.

**FIGURE 4:**
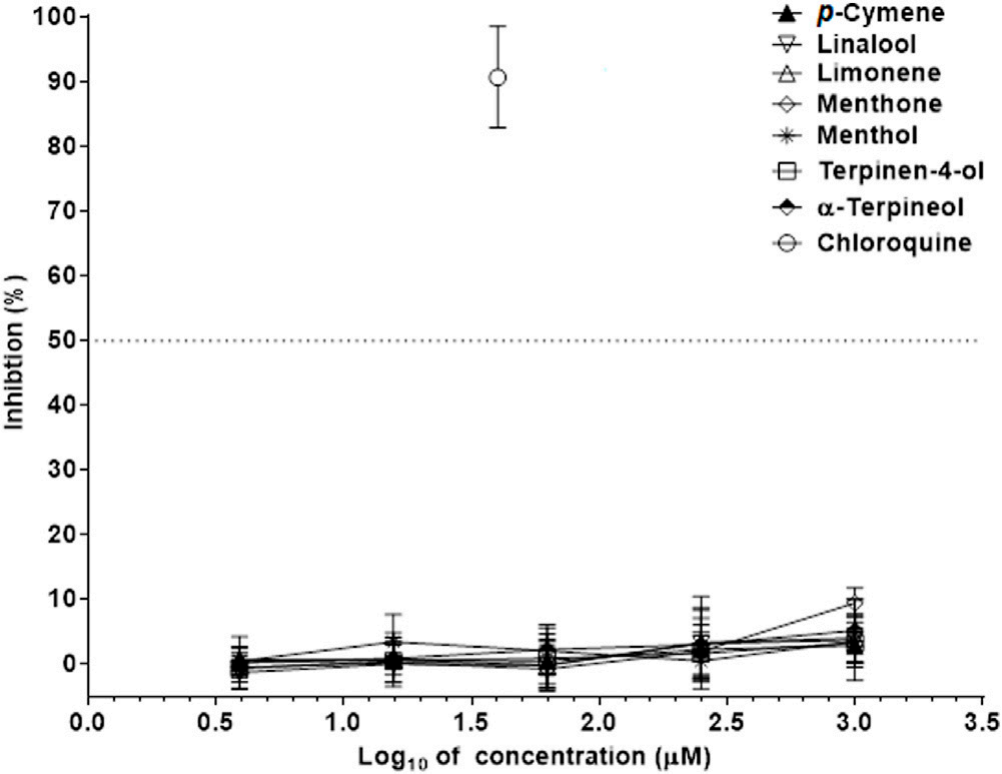
Absence of activity against SARS-CoV-2 in *p*-cymene, limonene, linalool, menthol, menthone, terpinen-4-ol, and α-terpineol. The inhibition rates of SARS-CoV-2 of monoterpenes in infected Vero cells were determined in 72 h incubation assays. Chloroquine was used as a reference antiviral compound. Data points represent the means (±SD) of three independent biological replicates with three technical replicates.

In the infected Vero 76 cells, monoterpenes inhibited the SARS-CoV2 in a dose-dependent manner (Figure 3). Chloroquine was used as a reference compound (Supplementary Figure S1). Carvone and carvacrol had the EC_50_ values against SARS-Cov2 smaller than concentration values of 100 μM (Table 3), which corresponds to those values of compounds selected as hits in drug discovery. Furthermore, as their EC_50_ values against SARS-Cov2 were significantly smaller than those of the other three active monoterpenes including, menthofuran, 1,8-cineol, and pulegone, their efficacy against SARS-CoV-2 was greater (Table 3). In addition, thymol had limited antiviral activity and inhibited the SARS-Cov-2 at the rate of 58.54 ± 10.45% (Figure 4). Seven monoterpenes including, *p*-cymene, limonene, linalool, menthol, menthone, terpinen-4-ol, and α-terpineol did not inhibit the SARS-CoV-2 at any significant rate (Table 3; Figure 4).

**FIGURE 5:**
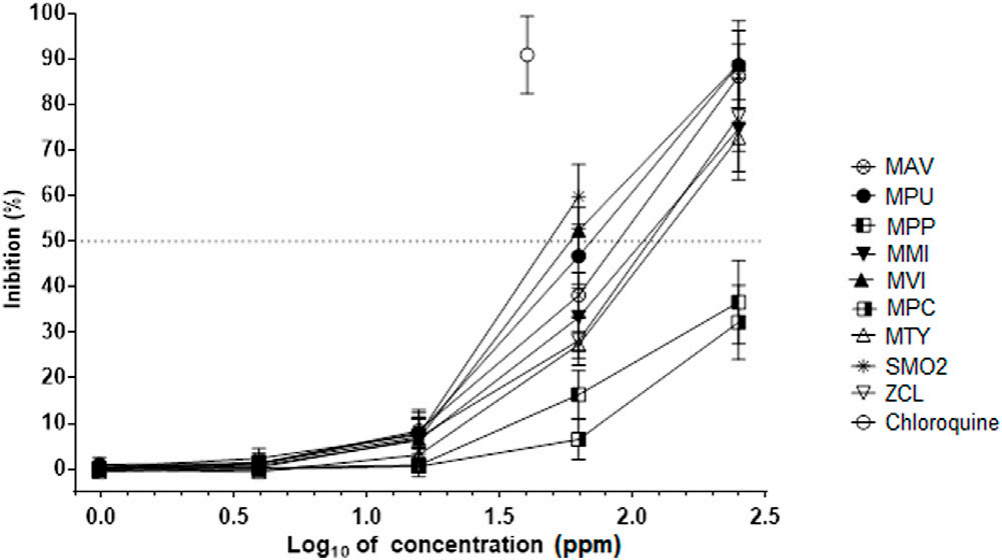
Inhibition of SARS-CoV-2 by essential oils. MAV—*Mentha aquatica* “Veronica”; MPU—*Mentha pulegium*; MPP—*Mentha x piperita* “Perpeta”; MMI—*Mentha microphylla*; MVI—*Mentha vilosa*; MPC—*Mentha x piperita* “Citrata”; SMO2—*Satureja montana*; MTY—*Micromeria thymifolia*; ZCL—*Ziziphora clinopodioid*es. The inhibition rates of SARS-CoV-2 of essential oils in infected Vero 76 cells were determined in 72 h incubation assays. Chloroquine was used as a reference antiviral compound. Data points represent the means (±SD) of three independent biological replicates with three technical replicates.

**FIGURE 6:**
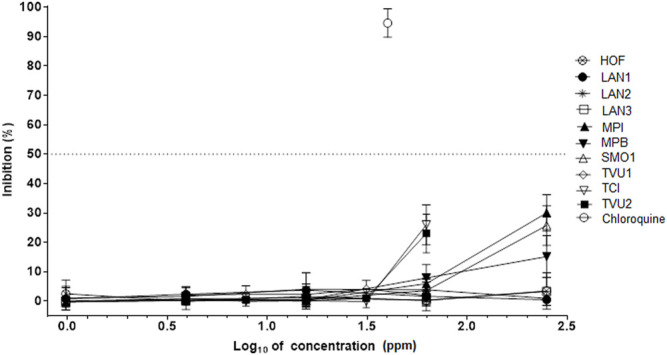
Absence of activity against SARS-CoV-2 in some essential oils. HOF—*Hyssopus officinalis*; LAN1—*Lavandula augustifolia* 1; LAN2—*Lavandula augustifolia* 2; LAN3—*Lavandula augustifolia* 3; MPI—*Mentha x piperita*; MMB—*Mentha x piperita* “Bergamot”; SMO1—*Satureja montana*; TVU1—*Thymus vulgaris* 1; TVU2—*Thymus vulgaris*; TCI—*Thumys x citriodorus*. The inhibition rates of SARS-CoV-2 of *essential oils* in infected Vero cells were determined in 72 h incubation assays. Chloroquine was used as *a* reference antiviral compound. Data points represent *the* means (±SD) of three independent biological replicates with three technical replicates.

Marked differences were found among nineteen tested essential oils in antiviral activity against SARS-Cov-2 in the infected Vero 76 cells. Six different essential oils including *M. aquatica* cv. Veronica (MAV), *M. pulegium* (MPU), *M. microphylla* (MMI), *M. x villosa* (MVI), *M. thymifolia* (MTY), and *Z. clinopodioides* (ZCL) inhibited the SARS-CoV-2 in a dose-dependent manner (Figure 5). As the EC_50_ value of *M. x villosa* essential oil was significantly smaller than that value of the other five active oils (Table 3), its efficacy against SARS-CoV-2 was the greatest. The other thirteen essential oils were inactive against SARS-Cov-2 (Figure 6; Table 3).

**FIGURE 7:**
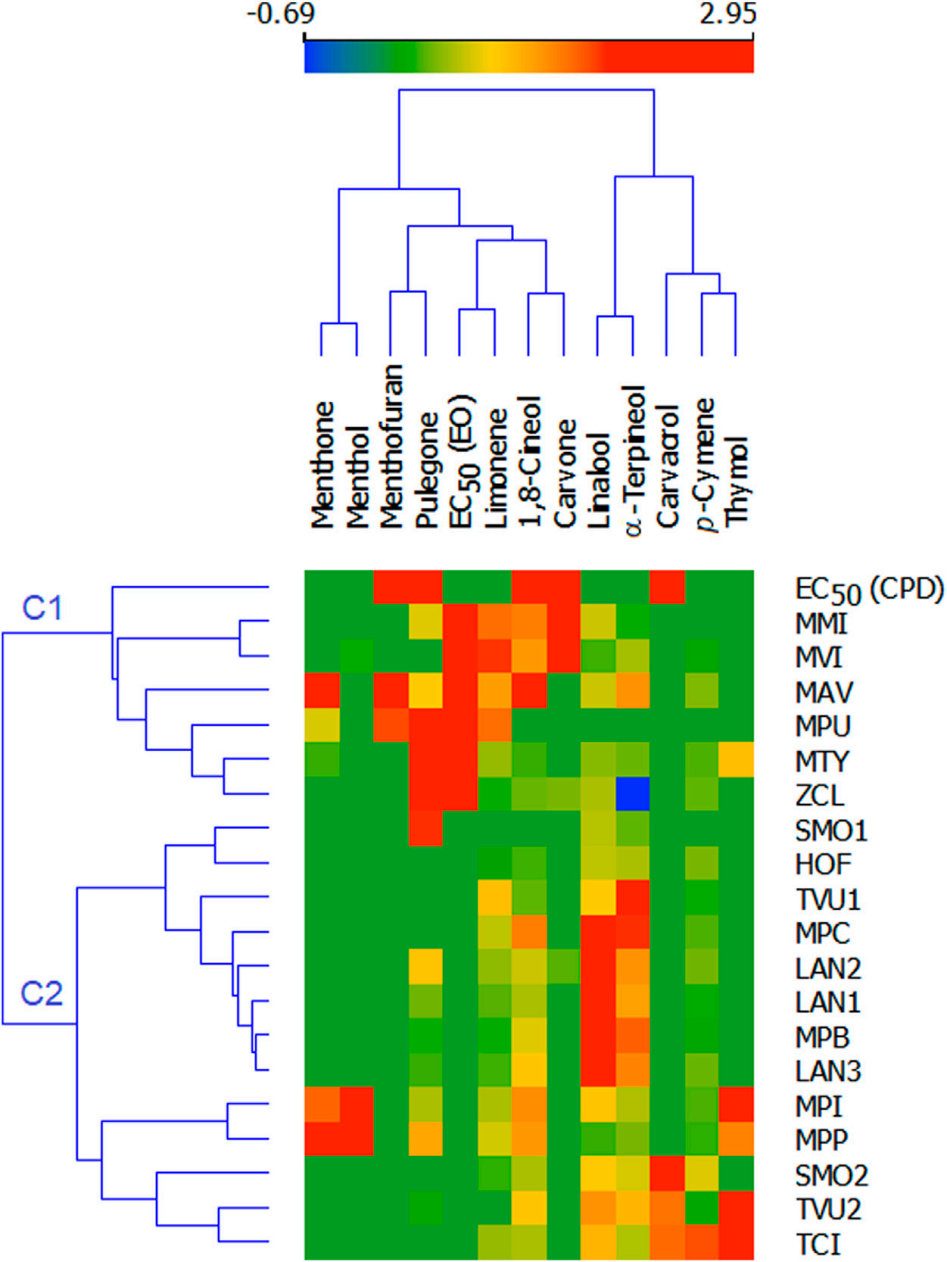
Heatmap of antiviral activity of selected monoterpenes and essential oils against Sars-Cov-2. MAV—*Mentha aquatica* “Veronica”; MPU—*Mentha pulegium*; MMI—*Mentha microphylla*; MVI—*Mentha vilosa*; MPI—*Mentha x piperita*; MPB—*Mentha x piperita* “Bergamot”; MPP—*Mentha x piperita* ‘Perpeta’; MPC—*Mentha x piperita* “Citrata”; TVU1—*Thymus vulgaris* 1; TVU2—*Thymus vulgaris 2*; TCI—*Thumys x citriodorus*; LAN1—*Lavandula augustifolia* 1; LAN2—*Lavandula augustifolia* 2; LAN3—*Lavandula augustifolia* 3; SMO1—*Satureja montana* 1; SMO2—*Satureja montana* 2; MTY—*Micromeria thymifolia*; HOF—*Hyssopus officinalis*; ZCL—*Ziziphora clinopodioides*. EC_50_ (CPD)—effective antiviral activity of single compound; EC_50_ (EO)—effective antiviral activity of essential oil.

For better visualization of differences in antiviral properties among essential oil, the heatmap of logarithmic values of antiviral activities of both essential oils and single monoterpenes was created (Figure 7). Essential oils with antiviral activity are grouped into cluster C1, while the inactive oils were grouped into cluster C2. The strong influence in clusterization had the presence of particular monoterpenes and their activities than the activities of essential oils. Strikingly, the oils that contain a high amount of carvone, pulegone, and carvacrol are grouped in an active cluster (several mints, *Z. clinopodioides* and *M. thymifolia*) are active against SARS-Cov2 while the essential oils containing significant concentrations of non-active monoterpenes (limonene, linalool) are separated as inactive (for example, *L. augustifolia* oils).

In addition, the structure-activity relations (SARs) were observed for monoterpenes in structurally related aromatic monoterpenes including *p*-cymene, thymol, and carvacrol, and aliphatic monoterpenes including limonene, carvone, terpinen-4-ol, and α-terpineol (Figure 1). The initial SAR analysis indicates the importance of the presence of hydroxyl group in monoterpenes with a benzene ring and the presence of the keto group in aliphatic monoterpenes.

## Discussion

### Essential Oil Composition

Among all medicinal and aromatic plants growing in the experimental fields of Crop Research Institute, for analyses of the bioactivity of essential oils against SARS-Cov-2 virus, we have selected the nineteen representative members of the Lamiaceae family that have a long tradition of use, both in folk medicine and the pharmaceutical and food industry (Tucker, 2007; Sharifi-Rad et al., 2017). Among mint species, *Mentha x piperita* L., *M. pulegium* L., and *M. spicata* L. are the most commercially important plants (Tucker, 2007). They have a long history of use in food flavoring and as therapeutic agents (Salehi et al., 2018; Mamadalieva et al., 2020). The use of thyme dates back to ancient Egypt where it was used as a flavoring agent for embalming (Zarzuelo and Crespo, 2002). The species of this genus are traditionally used in treating wounds, dermatitis, seborrhea, as a sedative, but also for the treatment of respiratory tract disorders. They possess antimicrobial activities against a wide array of bacteria and fungi (Ćavar Zeljković and Maksimović, 2015). The first records of the medicinal use of lavender date to ancient Greece. Historically, these species were used in veterinary practice, but also for human chest infections, as an active ingredient for massage of stiff joints, and also as an anti-repellent against mosquitoes. Nowadays, lavender species are mainly used in perfumery (Castle and Lis-Balchin, 2002). Many members of the genus *Satureja* are used as flavoring agents in the food, pharmaceutical, and cosmetic industries. Also, they have traditionally been used as a muscle pain reliever, tonic, and carminative agents to treat stomach and intestinal disorders such as cramps, nausea, indigestion, and diarrhea (Tepe and Cilkiz, 2016). In addition, *Hyssopus officinalis* is used for the treatment of cough, cold, loss of appetite, fungal infection, and spasmodic conditions (Sharifi-Rad et al., 2022), while *Ziziphora clinopodioides* is known as stomachic, carminative, and wound healing herb (Ozturk and Ercisli, 2007).

Essential oil compositions of the majority of these plant species have been already described in our previous studies (Ćavar et al., 2008; Ćavar Zeljković and Maksimović, 2015; Ćavar Zeljković et al., 2021b). However, it is well known that the essential oil composition is strongly dependent on environmental conditions and different chemotypes of essential oils can be detected from one plant species (Ćavar et al., 2008). Some of the selected plants, i.e., *L. augustifolia*, *T. vulgaris*, and *S. montana* exhibit high chemodiversity in essential oils (Slavkovska et al., 2001), and therefore, we have decided to test their different chemotypes. Most importantly, since the bioactivity of these oils against SARS-CoV-2 virus was the main focus of this study, it was necessary to evaluate their chemical compositions and compare them with the results from bioassays.

We have already studied the phytochemical profiles of *Mentha* species investigated here (Ćavar Zeljković et al., 2021b), and their phytochemical profiles presented here resembled them with small differences. Generally, these oils are having the same chemotype, though with slightly different levels of particular monoterpenes (Supplementary Table S1) which might be attributable to different reasons including the environmental conditions and the storage duration of harvested plants. However, the exact concentrations of their monoterpenes are reported here for the first time (Table 1). The essential oils of *M. pulegium* (MPU), *M. x piperita* cv. Perpeta (MPP), and *M. x villosa* (MVI) had pulegone, menthone, and menthol significantly higher than the concentration of 100 mg/ml, respectively. Such high concentrations were also observed for carvone in the essential oils of *M. x piperita* (MPI) and *M. x piperita* cv. Bergamot (MPB), and for linalool in the essential oils of *M. x piperita* cv. Bergamot (MPB) and *M. x piperita* cv. Citrata (MPC). In contrast, the essential oil of *M. aquatica* cv. Veronica (MAV) showed the highest heterogeneity among all mints, where none of the most abundant compounds did reach the concentration of 100 mg/ml of the oil.

Essential oils of both garden thyme (*Thymus vulgaris*) and winter savory (*Satureja montana*) are characterized as plants with high chemical polymorphism, even within a single population, and especially in populations coming from distant habitats (Slavkovska et al., 2001). Two chemotypes of *T. vulgaris* were investigated here, i.e., one with α-terpinyl acetate as the major compound (TVU1), and the other one with thymol as the most abundant volatile compound (TVU2) (Table 1). Thymol chemotype is the most common for this species, especially if grown in the Mediterranean area, followed by *p*-cymene or γ-terpinene chemotypes (Ćavar Zeljković and Maksimović, 2015). This is not surprising as *p*-cymene and γ-terpinene are, in fact, biochemical precursors of thymol and carvacrol. However, α-terpinyl acetate chemotype (Supplementary Table S1) is not very common for *T. vulgaris* but it is found in many other members of *Thymus* genus (Vaiciulyte et al., 2021). Also, two chemotypes of *S. montana* showed distinctions in monoterpene profiles. One chemotype (SMO1) is also uncommon for *S. montana* and contains 36.4% pulegone and 14.1% piperitenone, but it was described previously (Perrino et al., 2021), while the other chemotype (SMO2) is carvacrol chemotype that is quite common for the Mediterranean area (Table 1) (Slavkovska et al., 2001; Vidic et al., 2009). Interestingly, the essential oil from *M. thymifolia*, which is also known as *Satureja tymbra* L. or *Satureja thymifoilia* Scop., has a very similar composition as the SMO1 chemotype (Supplementary Table S1), which is in agreement with the literature (Šavikin et al., 2010).

The monoterpene profile of essential oils of *Z. clinopodioides* and *H. officinalis* was similar to those of previous studies. The oil of *Z. clinopodioides* was rich in pulegone (Baser et al., 1991), but it also contained significant levels of menthol derivatives. The oil of *H. officinalis* is very rich in pinocamphone derivatives as in a previous study by Zawislak (2016). Furthermore, it was also noted all three essential oils of *L. angustifolia* were linalool/linalyl acetate chemotypes, as described before (Dušková et al., 2016), but with significant differences in their major components (Supplementary Table S1).

### Antiviral Activity of Essential Oils Their Components Against SARS-Cov2

The findings presented here showed for the very first time that plant monoterpenes and essential oils as natural mixtures of monoterpenes might have significant antiviral properties against SARS-CoV-2 virus. Previous studies showed that both essential oils from different members of Lamiaceae plant species and their monoterpene components have activity against different (Das et al., 2021; Wani et al., 2021). Furthermore, *in silico* studies also showed that different monoterpenes from essential oils including, carvone, carvacrol, thymol, pulegone, and 1,8-cineol might have antiviral activity against SARS-CoV-2 (Silva et al., 2020; Das et al., 2021; Kim, 2021). Most significantly, since essential oils from six different plants, MAV, MPU, MMI, MVI, MTY, and ZCL (Figure 5; Table 3), and five different monoterpenes including carvone, carvacrol, menthofuran, 1,8-cineol, and pulegone (Figure 3; Table 2) inhibited the SARS-Cov-2 in the infected Vero 78 cells, their antiviral activity against this virus was demonstrated. These findings are intriguing and suggest that both the bioactive essential oils and single monoterpenes could be used in the development of formulations for different dietary and therapeutic measures against COVID-19, particularly in topical applications on respiratory tract mucosa, where biologically relevant concentrations could be achieved.

Our heatmap analysis of essential oils activity revealed that the antiviral property of essential oils varied as the essential oils with antiviral activity against SARS-CoV-2 from six different plants including MAV, MPU, MMI, MVI, MTY, and ZCL clearly separate as a cluster from the inactive essential oils (Figure 7). However, there is a trend in clusterization as the stronger influence in the grouping of essential oils had the presence of particular monoterpenes and their antiviral activities than the activities of essential oils as it is clearly shown that the oils containing significant concentrations of carvone, pulegone, and carvacrol (several mints, *M. thymifolia*, and *Z. clinopodioides*) are active against SARS-Cov2 while the essential oils that contain non-active monoterpenes (limonene, linalool) are inactive (Figure 7). Furthermore, such a trend was also observed in the differences in antiviral properties among active essential oils as the essential oils of MAV, MPU, MMI and MVI exhibited mild activity against SARS-Cov-2 (IC_50s_ < 200 ppm, Table 3) while the essential oils of MTY and ZCL showed quite weak activity (IC_50s_ > 200 ppm, Table 3). These differences in antiviral activity among the active essential oils also correlated with the differences in the efficacy of their single monoterpene components as it was observed that two monoterpenes carvacrol and carvone exhibited significant activity (IC_50s_ < 100 μM, Table 2), while 1,8-cineol, menthofuran, and pulegone revealed mild activity (IC_50s_ > 100 μM, Table 2). Subsequently, the essential oil of MVI with the highest carvone concentration (>200 mg/ml, Table 1) had the greatest antiviral activity against SARS-CoV-2 (Table 3). In addition, when the concentration of active monoterpenes was correlated with the activity of the essential oils, carvone revealed the highest Pearson coefficient (r = 0.557), following menthofuran, and 1,8-cineol.

Previous *in silico* studies showed that the monoterpenes used in this study and other common essential oil constituents can bind and interfere with the function of viral proteins including main protease, endoribonuclease, ADP ribose phosphatase, RNA-dependent RNA polymerase, and spike protein, and human proteins important for viral life cycle including angiotensin-converting enzyme (Silva et al., 2020; Torres Neto et al., 2021). Reported docking values of monoterpenes (Silva et al., 2020) used in this study for binding the aforementioned protein targets are in the agreement with their antiviral activity against SARS-CoV-2 (Table 2) with one exception as their results suggest that limonene should have the best antiviral activity, which, is not the case in our study. However, the molecular docking study of Panikar et al. (2021) shows that limonene inhibits SARS-Cov-2 protease three times weaker than 1,8-cineol, which is shown here to have antiviral activity. Furthermore, while the molecular docking study of Istifli et al. (2020) analyzed the binding energy of monoterpenes used in this study to spike protein and the cellular proteases, transmembrane serine protease 2, cathepsin B, and cathepsin L and showed that the most effective monoterpenes in inhibiting these proteins are carvone and carvacrol, which is in agreement with their antiviral activity against SARS-CoV-2 virus in this study. Our SARs analyses also revealed that the keto group of carvone and the hydroxyl group of carvacrol are associated with antiviral activity, and future studies on their more effective derivatives should focus on these positions.

As the monoterpenes of essential oils are extracted from plant tissues together and act in the mixture where both additive and synergistic effects among them can occur (Szczepaniak et al., 2011; Scandorieiro et al., 2016; Sharma et al., 2020), there could be even some synergistic effect among them in activity against SARS-Cov-2. However, in this study, this might be only the case in the more diverse essential oils of MVA and MPU where synergy might occur between 1,8-cineole, pulegone, and menthofuran. On the other hand, such interactions among monoterpenes could increase their cytotoxicity to human cells (Bassolé and Juliani, 2012). Our results also showed that the three monoterpenes, i.e., carvacrol, menthofuran, and thymol, and the essential oils with their high content exhibited limited but significant cytotoxicity to Vero76 cells (*S. montana*, *T. vulgaris*, and *T. x citriodorus*; Tables 2, 3).

As the cytotoxicity to Vero 76 cells of some monoterpenes and essential oils was observed, for comparative analyses of their therapeutic prospects, the therapeutic indices (TI) were used both for active essential oils and their monoterpene components (Tables 2, 3). Essential oils that showed activity against SARS-Cov-2 revealed TI from 2.72 for *M. aquatica* cv. Veronica, to 9.27 and >13.34 for *M. x villosa* and *M. pulegium*, respectively (Table 3). Considering the monoterpenes, TI was ranging from >2.55 for pulegone to 6.23 for carvacrol (Table 2). These results can be directly corroborated with those of previous studies (Pauli and Schilcher, 2004; Wang et al., 2020), where such dosages of carvacrol, carvone, and other monoterpenes from the essential oils were found to be appropriate for inhalation therapy of viral respiratory infections.

## Conclusion

This study presents the very first report on *in vitro* analyses of the activity of nineteen different essential oils and their monoterpene components from plant species of the *Lamiaceae* family against the SARS-Cov2, which is associated with an ongoing global pandemic. Our analyses showed that the essential oils from *Mentha aquatica* L. cv. Veronica, *Mentha pulegium* L., *Mentha microphylla* K.Koch, *Mentha x villosa* Huds., *Micromeria thymifolia* (Scop.) Fritsch, and *Ziziphora clinopodioides* Lam.*,* and their monoterpene components, carvone, carvacrol, pelugone, menthofuran, and 1,8-cineole exhibited notable antiviral activity against SARS-CoV2. However, marked differences among monoterpenes and essential oils were found in antiviral activity. Carvone and carvacrol exhibited significant activity (IC_50_ < 100 μM) while pulegone, menthofuran, and 1, 8-cineole were less active. The SARs analyses showed that the antiviral activity of carvone and carvacrol are also associated with the presence of keto and hydroxyl groups, respectively. Furthermore, differences were also found among essential oils. The oil of *M. x villosa*, enriched with carvone, had the greatest antiviral activity among all tested essential oils while the essential oil of *M. thymifolia*, and *Z. clinopodioides* had the weakest antiviral activity among all active species (IC_50_ > 200 ppm). Such differences in antiviral activity correspond to efficiencies of their particular single monoterpenes. The presented results provide very important information on the antiviral activity of selected essential oils and their components that might be used in the measures against SARS-Cov-2. These essential oils have been used in various aspects of the pharmaceutical industry as topical antivirals, and their use might be extended in therapies of Covid-19.

## Data Availability

The raw data supporting the conclusion of this article will be made available by the authors, without undue reservation.

## Supplementary Material

Supplementary Figure S1 Activity of chloroquine against SARS-CoV-2. The inhibition rates of SARS-CoV-2 in infected Vero cells were determined in 72 h incubation assays. Data points represent means (± SD) of three independent biological replicates with three technical replicates.
