# Supplementary figures and images for: Antiviral Activity of Selected Lamiaceae Essential Oils and Their Monoterpenes Against SARS-Cov-2
Source: Front Pharmacol. 2022 May 2;13:893634. doi: 10.3389/fphar.2022.893634 (PMC9108200; doi:10.3389/fphar.2022.893634)

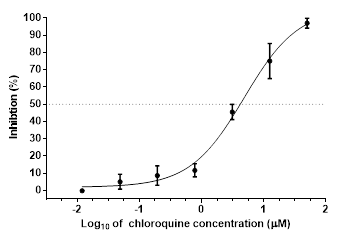

Supplement: Supplementary file 1 [file Image1.TIF]
