# Supplementary material for: Antiviral Activity of Selected Lamiaceae Essential Oils and Their Monoterpenes Against SARS-Cov-2
Source: Front Pharmacol. 2022 May 2;13:893634. doi: 10.3389/fphar.2022.893634 (PMC9108200; doi:10.3389/fphar.2022.893634)
Supplement: Supplementary file 2 [file Table1.DOC]

**Supplementary Table 1.** Chemical composition of investigated essential oils.

| **RI** | **Compound** | **MAV** | **MPU** | **MPI** | **MPB** | **MPP** | **MMI** | **MVI** | **MPC** | **TVU1** | **TVU2** | **TCI** | **LAN1** | **LAN2** | **LAN3** | **SMO1** | **SMO2** | **MTY** | **HOF** | **ZCL** |
| --- | --- | --- | --- | --- | --- | --- | --- | --- | --- | --- | --- | --- | --- | --- | --- | --- | --- | --- | --- | --- |
| 923 | α-Thujene | 0.1 |  |  |  |  |  |  |  |  |  | 1.0 |  |  |  |  |  |  |  |  |
| 929 | α-Pinene | 2.6 | 0.8 | 0.4 |  | 0.5 | 1.0 | 0.7 | 0.3 |  |  | 0.6 |  | 0.1 |  |  |  |  |  |  |
| 944 | Camphene | 0.5 |  |  |  |  |  |  |  | 0.1 |  | 0.5 |  | 0.1 |  |  |  |  |  |  |
| 968 | Sabinene | 1.5 | 0.3 | 0.2 |  | 0.2 | 0.7 | 0.4 | 0.6 | 1.5 |  |  |  |  |  |  |  |  |  |  |
| 972 | β-Pinene | 3.7 | 1.3 | 0.6 | 0.2 | 0.8 | 1.6 | 0.7 | 0.6 | 0.1 | 0.8 | 0.6 | 0.2 | 0.4 | 0.3 |  | 0.9 | 0.2 | 0.5 |  |
| 981 | 3-Octanone | 0.1 |  |  |  |  |  |  |  |  | 0.2 |  | 1.0 | 0.6 |  |  |  |  |  |  |
| 987 | β-Pinene | 0.1 |  |  | 0.2 |  | 0.3 |  | 0.8 | 0.9 |  | 1.1 |  | 0.2 | 0.1 |  | 0.1 |  |  |  |
| 990 | 3-Octanol |  |  | 0.1 |  | 0.2 | 0.6 | 0.5 |  |  | 0.3 |  | 0.2 | 0.2 |  |  |  | 0.2 |  |  |
| 1002 | α-Phellandrene |  |  |  |  |  |  |  |  |  |  | 0.2 |  |  |  |  |  |  |  |  |
| 1007 | Δ-3-Carene |  |  |  |  |  |  |  |  |  |  |  |  | 0.3 | 0.5 |  | 0.2 |  |  |  |
| 1013 | α-Terpinene |  |  |  |  |  |  |  |  |  |  | 1.4 |  |  |  |  |  |  |  |  |
| 1020 | *p*-Cymene | 0.6 |  | 0.5 | 0.2 | 0.3 |  | 0.2 | 0.3 | 0.2 | 0.2 | 13.2 | 0.4 | 0.5 | 0.5 |  | 1.0 | 0.5 | 1.1 | 0.5 |
| 1024 | Limonene | 3.8 | 6.7 | 0.9 | 0.3 | 1.1 | 7.5 | 13.4 | 0.8 | 1.4 |  | 0.4 | 0.3 | 0.4 | 0.2 |  | 0.1 | 0.5 |  | 0.1 |
| 1026 | 1,8-Cineol | 25.5 |  | 6.9 | 1.7 | 5.0 | 7.1 | 5.2 | 6.1 | 0.3 | 2.0 | 0.5 | 0.9 | 0.8 | 1.9 | 0.5 |  | 0.3 | 0.5 | 0.3 |
| 1032 | (*Z*)-β-Ocimene |  |  |  |  |  |  |  |  |  |  |  |  | 0.3 |  |  |  |  |  |  |
| 1043 | (*E*)-β-Ocimene |  |  |  |  |  |  |  |  |  |  |  |  | 0.1 |  |  |  |  |  |  |
| 1053 | γ-Terpinene |  |  |  |  |  |  |  |  |  | 0.8 | 10.1 |  |  |  |  | 2.6 |  |  |  |
| 1061 | *cis*-Sabinene hydrate |  |  | 1.6 |  |  |  |  | 0.1 |  | 0.8 | 0.6 |  | 0.1 | 0.1 |  | 0.4 |  | 2.8 |  |
| 1067 | *cis*-Linalool oxide |  |  |  |  |  |  |  |  |  |  |  |  |  |  | 0.4 |  |  | 0.9 | 0.1 |
| 1084 | *trans*-Linalool oxide |  |  | 0.2 | 2.8 |  |  |  | 0.3 |  |  |  | 3.3 | 0.6 | 0.7 |  | 0.1 |  |  |  |
| 1084 | Terpinolene |  |  |  |  |  |  |  |  |  | 0.2 |  |  |  |  |  |  |  |  |  |
| 1096 | Linalool | 0.6 |  | 1.9 | 43.4 | 0.1 | 0.8 |  | 36.2 | 1.0 |  | 1.5 | 39.1 | 50.0 | 35.7 | 1.7 | 0.7 | 0.3 | 0.8 | 0.4 |
| 1102 | *cis*-Thujone |  |  |  |  |  |  |  |  |  |  |  |  |  |  |  |  |  | 0.3 |  |
| 1108 | 1-Octen-3-yl acetate |  |  |  | 0.4 |  |  |  | 0.6 | 0.2 |  |  | 0.7 | 0.5 | 1.2 |  |  |  |  |  |
| 1120 | 3-Octanol acetate |  |  | 0.2 |  |  |  | 0.1 | 1.2 |  |  |  |  |  |  |  |  |  |  |  |
| 1133 | *trans*-Sabinol |  | 0.2 |  |  |  | 0.1 | 0.6 |  |  |  |  |  |  |  |  |  |  | 0.3 |  |
| 1139 | Camphor |  |  |  |  |  |  |  |  |  | 1.3 | 0.3 | 1.3 | 0.4 | 0.3 | 0.9 | 0.2 | 0.2 | 0.2 | 0.3 |
| 1143 | *p*-Menth-3-en-8-ol |  |  |  |  |  |  |  |  |  |  |  |  |  |  |  |  |  |  | 2.9 |
| 1148 | Menthone | 13.9 | 0.7 | 8.7 |  | 45.0 |  |  |  |  |  |  |  |  |  |  |  | 0.1 |  | 0.3 |
| 1155 | *trans*-Pinocamphone |  |  |  |  |  |  |  |  |  |  |  |  |  |  |  |  |  | 0.6 |  |
| 1157 | Pinocarvone |  |  |  |  |  |  |  |  |  |  |  |  |  |  |  |  |  |  |  |
| 1159 | *iso*-Menthone |  |  |  |  |  |  |  |  |  |  |  |  |  |  | 4.8 |  | 2.9 |  | 16.5 |
| 1159 | Menthofuran | 33.2 | 15.3 |  |  |  |  |  |  |  |  |  |  |  |  |  |  |  |  |  |
| 1160 | Borneol |  |  |  |  |  |  |  |  | 0.2 | 0.5 | 1.2 | 4.1 | 1.8 | 1.3 |  | 1.1 |  | 0.2 |  |
| 1160 | *neo*-Menthol |  |  |  |  | 14.1 |  |  |  |  |  |  |  |  |  |  |  |  |  |  |
| 1162 | δ-Terpineol |  |  |  |  |  | 0.2 | 0.1 |  |  |  |  |  |  |  |  |  |  |  |  |
| 1162 | Lavandulol |  |  |  |  |  |  |  |  |  |  |  | 0.2 | 2.1 | 0.7 |  |  |  |  |  |
| 1164 | *trans*-Linalool oxide |  |  |  | 0.3 |  |  |  |  |  |  |  |  |  |  |  |  |  |  |  |
| 1167 | Menthol |  |  | 53.8 |  | 19.2 |  | 0.1 |  |  |  |  |  |  |  |  |  |  |  |  |
| 1168 | *cis*-Pinocamphone |  |  |  | 0.3 |  |  |  |  |  | 0.5 |  | 0.3 | 0.1 | 0.1 | 0.9 | 0.2 | 0.9 | 63.3 | 0.2 |
| 1172 | Terpinen-4-ol | 0.3 | 0.6 | 0.5 |  |  |  |  | 0.3 | 0.1 | 1.0 | 0.5 | 1.9 | 7.4 | 2.9 | 0.6 | 0.7 |  | 0.3 | 0.5 |
| 1177 | *p*-Cymen-8-ol |  |  |  |  |  |  |  |  |  |  |  |  |  | 0.1 |  |  |  |  |  |
| 1179 | *iso*-Menthol |  |  | 0.4 |  | 0.3 |  |  |  |  |  |  |  |  |  |  |  |  |  | 16.1 |
| 1180 | Cryptone |  |  | 1.3 |  | 0.2 |  |  |  |  |  |  |  |  |  |  |  |  |  |  |
| 1184 | *neoiso*-Menthol |  |  |  |  |  |  |  |  |  |  |  | 0.6 | 0.7 | 0.2 |  |  |  |  | 0.5 |
| 1185 | α-Terpineol | 1.8 |  | 0.3 | 5.1 | 0.1 | 0.9 | 0.3 | 5.2 | 13.4 | 0.3 | 0.2 | 1.9 | 1.7 | 1.9 | 0.4 | 0.2 | 0.1 | 0.4 | 0.1 |
| 1189 | Hexyl butanoate |  |  |  |  |  |  |  |  |  |  |  | 0.2 | 0.2 | 0.2 |  |  |  |  |  |
| 1191 | Myrtenal |  |  |  |  |  |  |  |  |  |  |  |  |  |  |  |  |  | 0.9 |  |
| 1192 | *cis*-Dihydro carvone |  |  |  |  |  | 18.2 | 7.2 |  |  | 0.1 |  |  |  |  |  |  |  |  |  |
| 1199 | *trans*-Dihydro carvone |  |  |  |  |  | 0.6 | 0.1 |  |  |  |  |  |  |  |  |  |  |  |  |
| 1214 | *trans*-Carveol |  | 0.1 |  |  |  | 0.2 | 0.4 |  |  |  |  |  |  |  |  |  |  |  |  |
| 1230 | Thymol methyl ether |  |  |  |  |  |  |  |  |  | 2.3 | 2.1 |  |  |  |  |  |  |  |  |
| 1233 | Pulegone | 1.3 | 27.1 | 0.5 | 0.2 | 3.4 | 0.4 |  |  |  | 0.9 |  | 0.2 | 1.5 | 0.2 | 36.4 | 0.3 | 55.3 |  | 41.3 |
| 1238 | Carvone |  |  |  |  |  | 55.4 | 60.1 |  |  |  |  |  | 0.1 |  |  |  |  |  |  |
| 1239 | Carvacrol methyl ether |  | 0.2 |  |  |  |  |  |  |  | 3.5 | 1.3 |  |  |  |  |  |  |  |  |
| 1242 | *trans*-2-Hydroxy-pinocamphone |  |  |  |  |  |  |  |  |  |  |  |  |  |  |  |  |  | 6.2 |  |
| 1248 | Piperitone |  | 8.1 | 0.7 |  | 0.9 |  | 0.7 |  |  |  |  |  | 0.2 | 0.1 | 1.3 |  | 0.2 | 0.2 | 1.3 |
| 1252 | Linalool acetate |  |  | 1.7 | 29.4 |  | 0.4 |  | 27.9 |  | 0.4 |  | 25.7 | 19.4 | 36.2 | 1.0 | 0.3 | 0.1 | 0.7 | 0.2 |
| 1268 | *n*-Decanol |  |  |  | 0.1 |  |  |  |  |  |  |  | 0.2 | 0.1 |  |  |  |  |  |  |
| 1271 | *neo*-Menthyl acetate |  |  | 0.3 |  | 1.2 |  |  |  |  |  |  |  |  |  |  |  |  |  |  |
| 1273 | *trans*-Carvone oxide |  |  |  |  |  | 0.4 | 0.6 |  |  |  |  |  |  |  |  |  |  |  |  |
| 1280 | (*E*)-Anethole | 0.3 | 0.3 |  |  |  |  | 0.1 | 0.3 |  |  |  |  |  |  |  |  |  |  |  |
| 1281 | Isobornyl acetate |  |  |  |  |  |  |  |  |  |  |  |  |  |  | 0.5 |  |  |  |  |
| 1288 | Lavandulyl acetate |  |  |  | 0.2 |  |  |  |  |  |  |  | 3.4 | 3.7 | 4.3 | 0.4 |  |  | 0.3 | 0.2 |
| 1290 | Thymol |  |  | 7.0 |  | 2.4 |  | 0.2 |  |  | 75.4 | 58.5 |  |  |  |  | 0.3 | 0.8 |  |  |
| 1298 | Carvacrol |  |  |  |  |  |  |  |  |  | 2.1 | 1.8 |  |  |  | 0.7 | 88.2 | 0.6 | 0.6 | 0.5 |
| 1301 | Geranyl formate |  | 0.1 |  |  |  |  |  |  |  |  |  |  |  |  |  |  |  |  |  |
| 1302 | Terpinen-4-ol acetate |  |  | 0.4 |  | 0.2 |  | 0.3 |  |  |  |  |  |  |  |  |  |  |  |  |
| 1307 | *neo*-Dihydrocarveol acetate |  | 0.1 |  |  |  |  |  |  |  |  |  |  |  |  | 1.2 |  |  |  | 0.1 |
| 1324 | *iso*-Dihydro carveol acetate |  |  |  |  |  | 0.7 | 1.9 |  |  |  |  |  |  |  |  |  |  |  |  |
| 1331 | Mentho thiophene | 0.1 |  |  |  |  |  |  |  |  |  |  |  |  |  |  |  |  | 0.4 |  |
| 1336 | Piperitenone |  | 27.1 |  | 0.7 |  | 0.3 | 0.2 |  |  |  |  | 0.5 | 0.3 | 0.1 | 14.1 |  | 12.6 |  | 1.2 |
| 1347 | α-Terpinyl acetate | 0.9 | 0.3 | 0.8 | 0.6 |  |  |  | 13.2 | 77.9 | 0.1 | 0.1 | 1.0 |  |  | 1.7 |  | 1.0 |  | 0.7 |
| 1349 | Thymol acetate |  |  |  |  |  |  |  |  |  | 0.1 | 0.1 |  |  | 0.3 |  |  |  | 0.3 |  |
| 1360 | Piperitenone oxide |  | 0.2 |  |  |  |  |  |  |  |  |  |  |  |  |  |  |  |  |  |
| 1361 | Neryl acetate |  |  |  | 1.6 |  | 0.2 |  | 1.0 |  |  |  | 0.3 | 0.4 | 0.5 | 3.1 |  | 4.5 |  | 0.1 |
| 1368 | Carvacrol acetate |  | 0.2 |  | 0.1 | 0.1 | 0.2 | 0.3 |  |  |  |  |  |  |  |  | 0.2 | 0.2 |  | 0.2 |
| 1380 | Geranyl acetate |  |  |  | 2.8 |  |  |  | 1.9 |  |  |  | 0.6 | 0.6 | 0.9 |  |  |  |  |  |
| 1393 | (*Z*)-Jasmone |  | 0.1 |  | 0.2 |  |  |  |  |  |  |  | 0.3 |  |  |  |  |  |  |  |
| 1412 | (*E*)-Caryophyllene | 0.8 |  |  |  |  |  |  | 0.5 | 0.4 | 1.7 | 1.2 | 0.2 | 1.4 | 1.3 |  | 1.0 |  |  |  |
| 1414 | α-Santalene |  |  |  |  |  |  |  |  |  |  |  | 0.2 |  |  |  |  |  |  |  |
| 1453 | (*E*)-α-Farnesene |  |  |  |  |  |  |  |  | 0.1 |  |  |  | 0.5 | 0.6 |  |  |  |  |  |
| 1474 | γ-Muurolene | 0.1 |  |  |  |  |  |  |  | 0.1 |  | 0.1 |  |  |  |  | 0.1 |  |  | 0.1 |
| 1489 | Bicyclogermacrene | 5.3 | 0.1 | 0.9 |  | 2.7 |  |  |  |  |  |  |  |  |  |  |  | 0.3 |  | 0.3 |
| 1503 | β-Bisabolene |  |  |  |  |  |  |  |  |  |  |  |  |  |  |  | 0.2 |  |  |  |
| 1507 | γ-Cadinene |  |  |  | 0.1 |  |  |  |  |  |  | 0.1 |  |  | 0.4 |  | 0.1 |  |  |  |
| 1517 | δ-Cadinene | 0.3 |  |  |  |  |  |  |  |  |  | 0.1 |  |  |  |  | 0.1 |  |  |  |
| 1543 | Elemol |  |  |  |  |  |  |  | 0.3 |  |  |  |  |  | 0.1 |  |  |  | 0.6 |  |
| 1575 | Caryophyllene oxide | 0.1 | 0.8 | 0.6 | 0.8 | 0.2 | 1.1 | 1.1 | 0.2 | 0.3 | 0.8 | 0.4 | 2.2 | 1.1 | 3.3 |  | 0.1 |  | 1.0 |  |
| 1633 | α-Muurolol | 0.1 |  |  |  |  |  |  |  |  |  | 0.3 |  |  | 0.4 |  |  |  |  |  |
|  | Total identified | 97.6 | 90.6 | 91.6 | 91.9 | 98.1 | 98.7 | 95.5 | 98.4 | 98.0 | 96.2 | 99.8 | 91.1 | 98.8 | 97.7 | 70.4 | 99.1 | 81.7 | 83.4 | 85.0 |

RI – retention indices on HP-5MS UI column; MAV – *Mentha aquatica* ‘Veronica’; MPU – *Mentha pulegium*; MMI – *Mentha microphylla*; MVI – *Mentha vilosa*; MPI – *Mentha x piperita*; MPB – *Mentha x piperita* ‘Bergamot’; MPP – *Mentha x piperita* ‘Perpeta’; MPC – *Mentha x piperita* ‘Citrata’; TVU1 – *Thymus vulgaris* 1; TVU2 – *Thymus vulgaris 2*; TCI – *Thumys x citriodorus*; LAN1 – *Lavandula augustifolia* 1; LAN2 – *Lavandula augustifolia* 2; LAN3 – *Lavandula augustifolia* 3; SMO1 – *Satureja montana* 1; SMO2 – *Satureja montana* 2; MTY – *Micromeria thymifolia*; HOF – *Hyssopus officinalis*; ZCL – *Ziziphora clinopodioides*.
